# Prevalence of nonuse of helmet and seat belt and distracted driving on national highway in Pakistan 

**Published:** 2019-08

**Authors:** Ajmal Khan Khoso

**Affiliations:** ^*a*^ National Highways & Motorway Police, Pakistan.

## Abstract

**Background:**

Pakistan is a developing country with high number of road traffic injuries. High magnitude of road traffic injuries highlights deficiencies in road traffic laws which are not at par with current safety requirements resultantly fail to address important risk factors such as distracted driving, non-use of seat belt and helmet. Purpose: - To assess effect of deficient traffic laws related of distracted driving, seat belt and helmet usage in term of estimate prevalence of different types of distractions and use of helmet by motorcycle riders and pillion riders and seat belts by the drivers and the passengers on National Highway in Pakistan that can lead to road crashes and increasing the injuries.

**Methods:**

Roadside survey was carried on N-5 National Highway to estimate number of distracted drivers, use of helmet by riders and pillion riders and use of seat belt by drivers and passengers.

**Results:**

Three types of distraction were observed i.e. Mobile Phone distraction (24%); Equipment related distraction (1%) and eating related distraction (1%) amounting to 29% of drivers distracted ;car drivers (29%), other vehicle drivers (6%), motorcyclists (3%) and trailer drivers (3%). Likewise; 64% drivers were found without seat belt, LTV drivers (85%), car drivers (74%) and truck drivers (69%). Similarly; 97% passengers were found without seat belt, LTV passengers (100%), truck passenger (99%), cars passenger (97%), Oil tanker and trailer passenger (90%). Helmet violation was high with 87.4% motorcycle riders and 100% pillion riders were found without helmet.

**Conclusions:**

Lack of enforcement due to deficient traffic laws result in RTIs in Pakistan. There is need to improve legislation on different forms of distractions, use of seat belt among passengers and helmet use by pillion riders; so that enforcement efforts can be made to address risk factors effectively to meet Decade of Action 2011-2020 targets.

**Keywords:**

Seat belt, Helmet, Distraction, Pakistan

